# Nurses’ job preferences for working in deprived areas in Tehran: a discrete choice experiment

**DOI:** 10.1186/s12960-023-00875-9

**Published:** 2023-11-27

**Authors:** Amirmohammad Haddadfar, Sara Emamgholipour, Mohsen Razani, Mohammad Hassan Salehnejad

**Affiliations:** 1 Department of Health Management and Economics, Tehran University of Medical Sciences, Tehran, Iran; 2 Department of Psychology, Islamic Azad University, Boroujerd, Iran

**Keywords:** Discrete choice experiment, Job preferences

## Abstract

**Background:**

In Iran, the issue of the nursing shortage and unequal distribution exist simultaneously. The shortage of healthcare workers is one of the most important concerns of the health systems. In addition, the disparity in the distribution of healthcare workers between large metropolises and remote or non-capital areas has become a serious concern and a top priority to address. We conducted this study to identify and create a sufficient understanding of the different financial and non-financial preferences of nurses for working in deprived areas.

**Methods:**

This research was carried out in June and April 2022. It was carried out in three major phases. The factors influencing the nurses' job preferences were first discovered using qualitative methods. The second phase was conducting a pilot study and determining the best design for discrete choice experiment scenarios. The last phase involved publishing the questionnaire to gather information. Data were analyzed (discrete choice analysis) using JMP Pro 16 software.

**Results:**

A desirable job for the participants (nurses) in this study would have a higher salary, work in a city, the Rasmi employment contract, a low workload, adequate workplace facilities, an appropriate work schedule, and 1 to 3 years spent on the assigned job to promote to a higher position. Willingness to pay (WTP) and the probability of selecting different attribute levels were also calculated and reported. For example, the highest amount of money that a nurse expected to be paid was for changing the geographical location of the workplace from a city to a deprived area. In this case, a nurse tends to receive 91.87 million IRR more to move from a city to a deprived area to work. This amount of money was by far the most among other WTPs.

**Conclusion:**

The results of this study indicated that nurses are willing to forego net income in exchange for other favorable characteristics of their working environment and conditions. This shows that a variety of actions are accessible to policymakers that can greatly enhance the working conditions for nurses. The WTP and the probability of selecting various attributes may help policymakers plan more effectively.

**Supplementary Information:**

The online version contains supplementary material available at 10.1186/s12960-023-00875-9.

## Background

It is clear that human resources are crucial to the healthcare system. Delivering healthcare services successfully depends on both an adequate supply of healthcare personnel and their proper distribution. The shortage of healthcare workers is one of the most important concerns of the health systems. In addition, the disparity in the distribution of healthcare workers between large metropolises and remote or non-capital areas has become a serious concern and a top priority to address [1, 2]. In particular, the nursing workforce is a key element in enhancing the healthcare system and providing efficient care. It can be difficult to recruit nurses and other medical professionals to work in rural or underserved areas of most countries. Specifically, low- and middle-income countries have struggled not just because of unequal distribution and a lack of healthcare employees, but also as a result of healthcare workers migrating to work in developed countries [3, 4].

The increase in the average age of working nurses and retirement, the migration to developed countries, the inefficiency of healthcare systems, the lack of suitable job opportunities for nurses, unsafe work environments, the lack of independence of hospitals to recruit staff, low salaries, the low social level, etc., are considered as possible reasons for increasing demand for nursing staff [3, 5, 6].

In Iran, the issue of the nursing shortage and unequal distribution exist simultaneously [7], and effective policies are needed to manage nursing human resources properly. While this study focuses on determining the incentive packages and identifying the job preferences of nurses, governments may be interested in other solutions as well. In Iran, the Ministry of Health and Medical Education (MoHME) is responsible for the training and distribution of human resources in the health sector. The MoHME took some actions in order to employ forces in underserved areas and compensate for the disproportionate distribution. For example, the MoHME expanded rural medical education, attracted and compelled rural students in the health professions to work in those areas for extended periods with special contracts, and dedicated some medical graduates to work in these areas in a special 2-year program. Despite such actions, there are still many problems in attracting and retaining human resources in underserved areas of Iran [8, 9].

In this regard, information on health worker preferences for job attributes can assist policymakers in deciding which strategies to pursue to attract a sufficient number of health workers to underserved areas. Discrete choice experiments (DCE) are an applied policy tool that can be used to elicit such preference information [10]. DCEs assist policymakers in determining a ranking of health providers’ preferences toward possible incentive packages in a way that each attribute’s value is comparable to another [11].

Various job attributes, including salary and financial settlements as well as non-financial considerations, including workplace culture and proximity to family, were discovered as influencing factors on employment decisions in prior discrete choice experiments relating to the retention of healthcare workers [4, 10, 12]. Due to the difference between currencies, it is difficult to make a comparison between attributes used in other studies. The purpose of this research is to identify and create a sufficient understanding of the different financial and non-financial preferences of nurses for working in deprived areas by determining the relative importance of each attribute, calculating the willingness to accept (also known as the willingness to pay—the marginal rate of substitution of nonmonetary attributes for attributes with monetary value), and determining the marginal probability of selecting one attribute level over another.

## Methods

### Discrete choice experiment

A choice experiment studies an individual’s preferences for a set of product or process (in the case of a service) attributes. Respondents are presented with sets of product attributes, called profiles. Each respondent is shown a small set of profiles, called a choice set or scenario and asked to select the preference that he or she most prefers. Each respondent is usually presented with several choice sets [13]. DCEs have become more commonly used in healthcare settings, primarily to value patient experiences and evaluate trade-offs between health outcomes and patient experiences [14]. DCE investigations can reveal which job qualities are more important and which are less important from the healthcare worker’s perspective. The policy importance of the resulting preferences may be determined not only by how strong the specific options are but also by how realistic they are from the views of policymakers and health workers, as well as by the labor market's context-specific characteristics [15].

### Selecting attributes and levels

In order to create and develop attributes and levels, a combination of different methods based on Helter and Borhler proposed framework was used [16]. Literature review, interviews, and rating of collected attributes. A scoping literature review was conducted and resulted in 11 papers that were analyzed in terms of their attributes and levels. Moreover, in order to create new attributes and adapt the collected attributes from the previous step, in-depth interviews with seven current nurses and experts in nursing were conducted. Finally, a list of potential attributes and levels was created, and nurses were asked to rate them according to their importance in job decisions. Eight attributes were finally included in the design of DCE. These attributes were the salary, the location of the job (location), the type of employment contract, workload, type of health facility, workplace facilities, work schedule, and expected time spent on the assigned job for promotion to a higher position. Except for the type of employment contract and expected time spent on the assigned job for promotion to a higher position, which were two attributes extracted through the interviews, the other six attributes were both mentioned in the literature review and the interviews. According to the national employment law of Iran [17], Rasmi is a sort of employment contract between two parties (mostly the government and its employees), in which the duration of this contract is permanent. Therefore, its duration is longer than the other two types of contracts. Peymani is a type of contract that is somewhere between Rasmi and Gharardadi in terms of the duration of the contract. Gharardadi is a temporary contract that might also be short (e.g., 3 months). Table 1 shows all attributes and levels that were used in this study. Moreover, all scenarios that were used in this study are shown in the supplementary information section (Additional file 1).

**Table 1:** Attributes and levels as the main factors affecting nurses' job selection

Attribute	Levels	Description
Salary	100 million IRR (250 USD) 150 million IRR (375 USD) 200 million IRR (500 USD)	
Location	City Rural (a deprived area)	
Type of employment contract	Gharardadi Peymani Rasmi	
Workload	Heavy Moderate Low	Heavy (in the special department, 4 patients per nurse, and in the normal department, 10 patients or more per nurse) Moderate (in the special department, 3 patients per nurse, and in the normal department, 8 to 9 patients per nurse) Low (in the special, 2 patients per nurse, and in the normal department, 5 to 7 patients per nurse)
Type of health facility	Hospital Clinic Health House	
Workplace facilities	Adequate Inadequate	Inadequate (no free transportation, no free meals on shift, break room without amenities) Adequate (free transportation is available, Free meals on shift, break room with standard amenities like microwave and refrigerator)
Work schedule	Appropriate Inappropriate	Appropriate (regular shifts, and the full cooperation of the supervisor in monthly planning according to individual conditions) Inappropriate (irregular shifts in the month, the presence of night shifts, working on holidays, and the lack of cooperation of the supervisor in the monthly planning according to the individual)
Expected time spent on the assigned job for promotion to a higher position	1 to 3 years 3 to 5 years More than 5 years	

### Design of the experiment and the questionnaire

Considering 5 attributes with 3 levels and 3 attributes with 2 levels, 3^5 * 2^3 = 1944 full factorial designs are required. Although a full factorial design can estimate all possible interactions, it is usually used when a small number of attributes and levels are available and there is a need to collect information about all possible interactions. JMP Pro 16 (JMP® Pro *16*. SAS Institute Inc., Cary, NC, 1989–2021) was used to construct the final design of choice sets (scenarios). The result of the pilot investigation was used to create a local D-optimal design. A local D-optimal design takes into account the prior on the mean but does not include any information from a prior covariance matrix [18]. The design consisted of 18 scenarios and was blocked into 3 versions with 6 scenarios each. Each scenario consisted of 2 profiles. The participants were required to answer one of the three versions randomly. In addition, there are different methods to test the internal validity of DCEs [19]. The within-set dominant profile was used as an internal validity test (internal consistency) in the design of the experiment. Thus, each version had seven scenarios instead of the previous six. The participants who failed the internal validity test were excluded from the data analysis. Moreover, the dominant choice set was not considered in the data analysis. The questionnaire consisted of two parts: the first part included the demographic and socioeconomic status of the respondent, and the second part included the scenarios. A pilot study was conducted prior to the final study to test the face validity, intelligibility, and acceptability of the questionnaire.

### Study population, sample size, and data collection

The study's subjects were nurses who worked in Tehran (The capital of Iran) and enrolled in the Iranian Nursing Organization (INO). It was done using simple random sampling. Additionally, INO collaborated on the study's execution. Each nurse who is registered with INO has a unique number. We randomly used this number to invite them to participate in the study. The questionnaire was published by the INO in June and July 2022. 700 nurses were invited and 243 nurses participated in this study (after excluding those who failed the internal validity test, *n* = 13). The following formula proposed by Johnson and Orme was used to obtain the study's sample size [20]:

$$\left( {nta/c} \right)\, > \,500.$$

The sample size required for the main effects depends on the number of choice tasks (*t*), the total number of respondents (*n*), the number of alternatives (*a*), and the largest number of levels for any of the attributes (*c*). In this study (*n*) equals 243 nurses, (*t*) equals 18, (*a*) equals 2, and (*c*) equals 3. The threshold was 2916 when these parameters were added to the formula above, exceeding the minimum threshold (500) recommended as a sign of adequate sample size in DCE investigations. The minimum number of participants using this formula is 42 (if one participant answers all scenarios). However, because the scenarios were divided into three versions (each participant answered one), the minimum number of participants must be 126.

### Statistical analysis

Random utility theory provides the theoretical foundation for the analysis of the DCEs data. The utility (*U*) associated with a particular job is made up of 2 components: the deterministic component *V*_*ni*_ (where *V* is a function of observable characteristics) and the unobservable component *ε*_*ni*_. The utility, *U*, to individual *n* associated with job *i* can be specified as [21]:

$$U_{n} = V_{n} + \varepsilon_{n} .$$

The beta (*β*) coefficients generated from the logit model in the equation can be used for two main purposes [21]:

- To determine whether Attributes are statistically significant.
- The direction of the signs of the coefficients also provides an examination of the theoretical validity of the DCE model, that is, whether the coefficients move according to economic theory or predict expectations or not.

The analysis took place using the Choice Platform of JMP Pro 16. The choice platform uses a form of conditional logistic regression to estimate the probability that a configuration is preferred. Unlike simple logistic regression, choice modeling uses a linear model to model choices based on response attributes and not solely on subject characteristics [13]. In the choice platform of JMP, the parameter estimates report gives estimates and standard errors of the coefficients of utility associated with the effects (attributes) listed in the term column of the platform. The choice statistical model is expressed as follows [13]:

Let *X*[*k*] represent a subject attribute design row, with an intercept.

Let *Z*[*j*] represent a choice attribute design row, without intercept.

Then, the probability of a given choice for the *k'*th subject to the *j'*th choice of *m* choices is:

$${P}_{i} \left[jk\right]= \frac{\mathrm{exp}({\beta}^{\prime}\left(X\left[k\right]\otimes Z\left[j\right]\right))}{\sum_{l=1}^{m}\mathrm{exp}({\beta }^{\prime}\left(X\left[k\right]\otimes Z\left[l\right]\right))},$$

where:

- $\otimes$ is the Kronecker row-wise product,
- the numerator calculates for the *j'th* alternative actually chosen,
- the denominator sums over the *m* choices presented to the subject for that trial.

### Ethical considerations

This paper was part of the Master thesis of Amirmohammad Haddadfar and received the ethical approval of the Research Ethics Committees of the School of Public Health & Allied Medical Sciences-Tehran University of Medical Sciences (approval ID: IR.TUMS.SPH.REC.1400.344). All participants also completed an informed consent form before participating in this study.

## Results

Based on the research objectives and the specified sample size, 243 nurses participated in the study. First, the individuals' descriptive statistics were examined. The majority of the study participants were 144 women (about 60%). The average age of the study participants was around 30 years. 55% of people were single. Only 16% of the participants were heads of households. Most of them were working in government hospitals. Most of the participants had a bachelor's degree. The four-person family was the most common type of family. The average work experience of nurses is about 6 years. 20% of the nurses participating in the study had experience working in deprived areas. Table 2 shows other descriptive information and statistics of the study participants.

**Table 2:** Descriptive information and statistics of the study participants

Characteristics	*N *= 243	%
Gender		
Female	144	(59)
Male	99	(41)
Age		
21–25	78	(32)
26–30	83	(34)
31–39	72	(29)
40	16	(6)
Marital status		
Single	136	(55)
Married	105	(43)
Divorced/widow	2	(1)
Children (among those who married)		
None		
1	49	(45)
2	33	(30)
3	19	(17)
More than 3	3	(2.8)
The head of the household	3	(2.8)
Yes	38	(16)
No	206	(84)
Education		
Bachelor of Science	202	(83)
Master of Science	37	(15)
PhD and higher	4	(2)
The type of health facility		
Public clinic	5	(2)
Private clinic	6	(2)
Public hospital	165	(68)
Private hospital	65	(27)
Health House	2	(1)
The job position		
Nurse (general wards)	143	(59)
Critical care nurse	81	(33)
Head nurse	10	(4)
Supervisor and higher	9	(4)
Work experience		
0–2	78	(32)
2–5	68	(28)
6–10	43	(18)
10	54	(22)
Experience in working in deprived areas		
Yes	50	(20)
No	193	(80)

### DCE analysis and logit model

Table 3 presents the result of the conditional logit model. The parameter estimation report and the standard errors of the utility coefficients related to the listed attributes are presented. Based on this model, different levels of the examined attributes change the utility for nurses. All attributes were statistically significant, except for the type of the health facility. The statistical significance of coefficients indicates that each attribute level affects the respondents' choice of scenarios. Willingness to pay has also been calculated to examine trade-offs between attribute levels. Willingness to pay (WTP) refers to the maximum amount that a person is willing to pay for a particular attribute. In other words, it is also possible to refer to the willingness to pay as the willingness to accept (WTA), which reflects the portion of their monthly salary that respondents are willing to forgo for one level of an attribute in exchange for another. For example, if we want a nurse to change the geographical location of his or her job from a city to a deprived area (a rural area) with the conditions mentioned as a reference and keeping other factors constant, we need to pay 91,430,000 IRR more to accept working in the given circumstance. This amount is interpreted in the same way as the work schedule. In this way, a person is willing to give up 42,580,000 IRR from his salary in order to have a suitable work schedule (changing from the inappropriate level to the appropriate level).

**Table 3:** The result of the conditional logit model and willingness to pay

Attributes and their levels	Coefficients	SE	*P*-value	95% CI	WTP	95% CI
Monthly salary (unit: 10 000 000 IRR)	0.185	0.025	< 0.001	0.137 to 0.235		
Location (ref: city)			< 0.001			
Rural	− 0.846	0.106		− 1.058 to 0.642	9.143	8.068 to 10.219
Type of employment contract (ref: Gharardadi)			< 0.001			
Peymani	− 0.008	0.047		− 0.1 to 0.084	− 1.664	− 2.623 to – 0.676
Rasmi	0.324	0.066		0.196 to 0.455	− 3.461	− 4.598 to – 2.325
Workload (ref: moderate)			< 0.001			
Heavy	− 0.442	0.099		− 0.637 to – 0.245	3.115	2.153 to 4.077
Low	0.307	0.104		0.104 to 0.514	− 0.932	− 2.135 to 0.27
Type of health facility (ref: clinic)			0.5			
Hospital						
Health House	0.068	0.059		− 0.049 to 0.185	− 0.375	− 1.202 to 0.451
	− 0.066	0.091		− 0.245 to 0.112	0.351	− 1.085 to 1.787
Workplace facilities (ref: inadequate)			0.01			
Adequate	0.108	0.043		0.025 to 0.466	− 1.169	− 1.94 to – 0.397
Work schedule (ref: inappropriate)			< 0.001			
Appropriate	0.394	0.036		0.324 to 0.466	− 4.258	− 5.322 to – 3.195
Expected time spent on the assigned job for promotion to a higher position (ref: 1 to 3 years)			< 0.001			
3 to 5 years	0.084	0.508		− 0.015 to 0.184	0.768	− 0.074 to 1.612
More than 5 years	− 0.3104	0.083		− 0.475 to – 0.149	2.898	1.777 to 4.019
AICc	1752.631					
BIC	1815.833					
−2Log Likelihood	1728.415					
−2Firth Log Likelihood	1652.097					

*SE* standard error, *WTP* willingness to pay, *CI* confidence interval, *AICc* Akaike information criterion (for small sample size), *BIC* Bayesian information criterion

As the type of health facility was insignificant, it was excluded from the model. Table 4 shows the new willingness to pay for the remaining attributes, which shows a slight difference from Table 3.

**Table 4:** Willingness to pay excluding the type of health facility from the model

Attributes and their levels	*P*-value	WTP	95% CI
Monthly salary (unit: 10 000 000 IRR)	< 0.001		
Location (ref: city)	< 0.001	9.187	
Rural			8.045 to 10.329
Type of employment contract (ref: Gharardadi)	< 0.001		
Peymani		– 1.57	– 2.587 to – 0.553
Rasmi		– 3.45	– 4.659 to – 2.224
Workload (ref: moderate)	< 0.001		
Heavy		3.091	2.072 to 4.109
Low		– 0.65	– 1.646 to 0.34
Workplace facilities (ref: inadequate)	< 0.001		
Adequate		– 0.998	– 1.736 to – 0.26
Work schedule (ref: inappropriate)	< 0.001		
Appropriate		– 4.491	– 5.426 to – 3.555
Expected time spent on the assigned job for promotion to a higher position (ref: 1 to 3 years)	< 0.001		
3 to 5 years		0.835	– 0.049 to 1.72
More than 5 years		2.828	1.689 to 3.968

The probability that someone will select level A as opposed to level B is known as the marginal probability (assuming the levels of other attributes are constant). The marginal probability of selecting attribute levels is displayed in Table 4. For instance, if all three profiles have the same attributes and levels except for the workload (the total probability of choosing the levels of an attribute is 1), the probability of selecting a profile with a heavy workload compared to the other two types of workload is 20%, the probability of selecting a profile with a moderate workload is 43%, and the probability of selecting a profile with a low workload is 36% (Table 5).

**Table 5:** Marginal probability of selecting attribute levels

Attributes and levels	Marginal probability
Location	
City	0.155
Rural	0.844
Type of employment contract	
Gharardadi	0.234
Peymani	0.319
Rasmi	0.445
Workload	
Low	0.363
Moderate	0.432
Heavy	0.204
Workplace facilities	
Adequate	0.553
Inadequate	0.446
Work schedule	
Appropriate	0.687
Inappropriate	0.312
Expected time spent on the assigned job for promotion to a higher position	
1 to 3 years	0.407
3 to 5 years	0.353
More than 5 years	0.238

### Subgroup analysis

The effect of age, gender, marital status, having children, and education on job preferences was investigated. There was no significant difference between single and married nurses, the presence of children in the family, or different educational degrees. Age has a significant effect on the willingness to pay for three attributes. Our findings indicated that with increasing age, the desirability of working in rural areas slightly increases and the willingness to pay for (or accept) working in these areas decreases. Age has a minor negative effect on willingness to pay for an appropriate work schedule. In comparison with a 22-year-old nurse, a 37-year-old nurse tends to forego 3.65 million IRR of his wage to work with an appropriate work schedule. In other words, younger nurses value working with an appropriate schedule slightly more than older nurses do. Table 6 shows WTP for three different ages. Furthermore, with increasing age, the utility of job promotion dramatically decreases. While a 22-year-old nurse anticipates receiving 4.766 million IRR for a promotion that has a wait time of more than five years, this amount for a 37-year-old nurse is only 0.29 million IRR. The difference between the two levels (three to five years and more than five years waiting for a promotion) is quite negligible and is just 0.04 million IRR. Gender has only a significant effect on one attribute (location). A male nurse tends to receive 16.177 million IRR (ceteris paribus) as a monthly salary to work in rural areas (6.177 million IRR more than working in a city), while a female nurse tends to receive 21.647 million IRR to do so. Female nurses tend to receive approximately twice the pay of male nurses to work in rural areas. Therefore, recruiting male nurses to work in rural settings would cost less.

**Table 6:** The effect of age and gender on the willingness to pay

	Age		WTP (age)			Gender (ref: male)		WTP (gender)	
Attributes and their levels	CF	*P*-value	Age = 22	Age = 30	Age = 37	CF	*P*-value	Male	Female
Monthly salary (unit: 10 000 000 IRR)	− 0.004	0.10				− 0.026	0.08		
Location (ref: city)									
Rural	0.027	0.01	9.753	9.211	8.5	− 0.136	< 0.01	6.177	11.647
Type of employment contract (ref: Gharardadi)		0.89					0.16		
Peymani	− 0.001		− 1.581	− 1.585	− 1.59	− 0.003		− 0.84	− 2.365
Rasmi	− 0.004		− 3.272	− 3.493	− 3.778	0.105		− 1.77	− 4.973
Workload (ref: moderate)		0.47					0.43		
Heavy	0.014		3.4	3.211	2.962	− 0.015		2.752	3.663
Low	− 0.01		− 0.693	− 0.558	− 0.383	0.008		− 0.743	− 0.989
Workplace facilities (ref: inadequate)		0.74					0.83		
Adequate	− 0.002		− 0.918	− 0.918	− 0.917	0.007		− 0.709	− 1.129
Work schedule (ref: inappropriate)		0.02					0.58		
Appropriate	− 0.012		− 4.837	− 4.678	− 4.472	0.02		− 3.421	− 5.079
Expected time spent on the assigned job for promotion to a higher position (ref: 1 to 3 years)		< 0.01					0.73		
3 to 5 years	− 0.011		1.252	0.815	0.25	− 0.034		2.288	1.178
More than 5 years	0.037		4.766	2.814	0.29	0.008		0.32	3.255

### Simulation of different scenarios

**Fig. 1:**
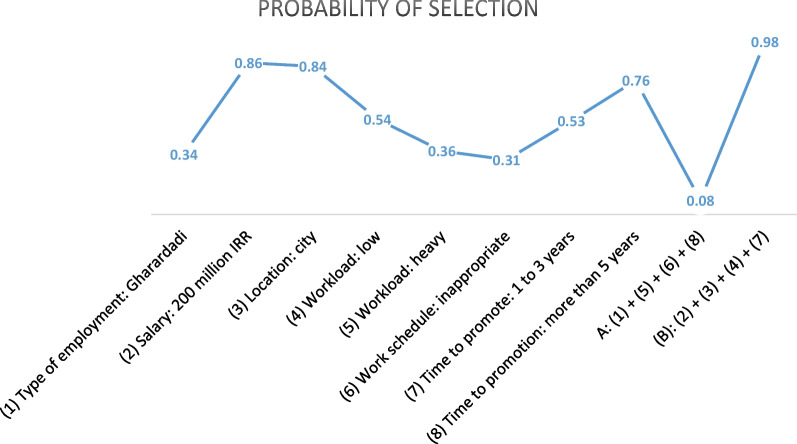
Simulation of the probability of choosing different scenarios with the reference: salary of 100 million IRR, location in a deprived area, type of employment contract (Rasmi), moderate workload, insufficient workplace facilities, an appropriate work schedule, and waiting between 3 and 5 years for promotion to a higher position

Preferences and potential possibilities for accepting a job under certain conditions, for example, the policy of attracting and retaining nurses to work in deprived areas, are simulated by the JMP. Some examples of different combinations of attributes and levels obtained from the simulation are shown in Fig. 1.

In this figure, a reference scenario (a random combination of different levels of attributes) has been set, all conditions (other levels) have remained constant (ceteris paribus), and only the one that is mentioned inside the diagram has changed. Finally, the probability of choosing a new scenario is compared with the reference scenario, and this probability is mentioned in the diagram. For example, with a change in the type of employment contract from Rasmi (reference scenario) to Gharardadi (scenario 1), the probability of choosing scenario 1 is 34%, while the probability of choosing the reference scenario is 66%. In the figure, (A) presents the least desirable scenario, and (B) presents the most desirable scenario. A large number of combinations of different levels were simulated by JMP using the results of this study, and Fig. 1 shows only a few of them.

## Discussion

Considering the importance of human resources as a vital part of the healthcare system and considerations related to the shortage and disproportionate distribution of human resources, this study was carried out. Likewise, a significant concern in Iran has been the unequal distribution of nurses. DCEs have been applied to research healthcare workers' preferences. This approach is frequently used to address the uneven distribution of health professionals. Policymakers can develop successful policies and identify incentives for health workers with the assistance of DCE investigations.

First, attributes and levels were defined using qualitative methods such as in-depth interviews and a review of the literature. Second, a pilot study was conducted. Based on the results of the previous stages, the final design of the choice sets was created. Finally, the collected data were analyzed using JMP.

The findings in our study were in agreement with previous research from other nations and showed that a health worker's preference for a job is highly influenced by both financial and non-financial factors [9, 22, 23]. Alongside other studies, respondents in this study preferred jobs that had higher salaries. The highest amount of money that a nurse expected to be paid was for changing the geographical location of the workplace from a city to a deprived area. In this case, a nurse tends to receive 91.87 million IRR more to move from a city to a deprived area to work. This amount of money was by far the most among other WTPs, which shows this attribute is the most important factor in nurses’ decision-making in this study. Moreover, the marginal likelihood of choosing a job in a city was about 84%, compared to the likelihood of choosing a job in a deprived area, which was roughly 16%. Out of the entire attribute levels examined in this study, this disparity in the probability of selecting levels was the greatest. Other studies have shown that health workers with a rural background are more likely to practice in rural areas after completing their studies [24]. According to our findings, relocating a job from a city to a deprived area would be expensive (the WTP for this relocation is nearly equal to a nurse's early-career monthly salary). Thus, attracting and retaining students from rural areas was recommended by other studies and it might be one strategy to reduce the costs [22, 24]. In addition, female nurses expect to receive higher salaries than male nurses when working in rural areas. In this regard, it is suggested that policymakers focus on attracting male nurses to work in rural settings, which would be less costly. Finally, with increasing age, the utility of working in rural areas increases, and according to Table 6, older nurses tend to receive lower salaries than younger nurses to work in rural areas. The workload was the other factor that affected participants' decisions. In this study, it referred to the number of patients to a nurse in a shift as an indicator of workload. Participants in this study were willing to accept working with a heavy workload in exchange for receiving 30.91 million IRR more (from a moderate workload to a heavy workload), while they were willing to forego only 6.5 million IRR of their monthly salary to work with a low workload (from a moderate workload to a low workload). In addition, a profile containing a heavy workload had a significantly lower chance of being selected by participants in comparison with the other two levels. A shortage in the number of health workers might be the cause of heavy workloads. As a result, having a sufficient number of healthcare workers along with proper distribution is critical. The attribute entitled "type of health facility" was not statistically significant. Our findings did not support prior research's conclusions that the type of healthcare facility matters to participants' decision-making [23]. In this regard, the differences between a hospital, a clinic, and a health house can be seen in terms of their workplace facilities, workload, location, etc., which could be one factor contributing to the lack of significance of the type of health facility. The three previously mentioned attributes and this one might overlap as we included them in the attributes of this study. The health house is the smallest unit providing primary care and is usually located in underprivileged regions (primarily in the south of Tehran). Conversely, the hospitals are well-equipped and typically located in the city center (not always). As a result, participants might not consider it while making a decision. Workplace facilities were also important in participants' decision-making. Other studies showed that improving the quality of facilities might be cost-effective [10]. Nurses were willing to forego 0.998 million IRR to work in a workplace with adequate facilities. The probability of selecting a job with adequate facilities over a job with inadequate facilities was roughly 55% to 45%. The difference between the selection probabilities of these attributes was the lowest among all two-level attributes in this study. Thus, it can be assumed that it is the least important attribute (factor) nurses consider when deciding to choose a job. The work schedule was the second-highest WTP after location. Participants tend to forego 4.491 million IRR of their monthly salary to have an appropriate work schedule. Furthermore, subgroup analysis showed that older nurses tend to receive 7% less salary than their younger counterparts in terms of working within an inappropriate work schedule. This may indicate that as nurses get older, they get more accustomed to the terms and circumstances of their work, which leads to a lower willingness to pay for this attribute. During the interviews, the interviewees emphasized during the in-depth interviews that the work schedule is one of the most crucial aspects of their jobs and that a poor work schedule can significantly affect their decisions regarding their careers. It was also claimed that an inappropriate work schedule would cause them to quit their position as a nurse. The results of our study confirmed this assertion. Apart from salary and location, this is considered a third key factor affecting the job preferences of nurses in this study. Selecting a job with the appropriate work schedule had a probability of around 69% while selecting one with an inappropriate schedule had a probability of 31%. These probabilities thereby demonstrate the significance of this attribute among nurses. Expected time spent on the assigned job for promotion to a higher position was an attribute that was subsequently created after in-depth interviews with nurses and experts. The probability of selecting a profile with a wait of more than 5 years was quite lower than the other two levels of this attribute (roughly 24%). This attribute shows that the future expectations of participants from their jobs were also important in their decisions. Moreover, our study showed that job promotion is highly important for young nurses in comparison with their older nurses, as they are willing to receive more money than their older counterparts when both of them have to wait longer for promotion. The utility of promotion is maximized in their 20 s and slowly decreases until their 40 s when it has limited utility for them. Based on our findings, policymakers might take into account a hierarchical promotion system that specifically encourages young nurses to work in underserved areas and keeps their motivation at a high level.

### Strengths and limitations

This study has a number of advantages. This study, to our knowledge, is the first to use a discrete choice experiment to examine the job preferences of nursing personnel in Iran despite the country's shortage and uneven distribution of nursing cadres. Second, subgroup analysis was carried out in addition to the simulation of the probability of selecting scenarios.

On the contrary, there were some limitations in this study. First, because the data collection was carried out online, a simple method of testing the internal validity (within-set dominated pairs) of the questionnaire was used. A simple method may decrease the accuracy of the internal validity test (Additional file 1). Second, the attribute type of health facility may overlap with other attributes of this study due to its statistical insignificance. Finally, the effect of participants’ urban or rural backgrounds on their decision-making to work in rural areas was not investigated in this study.

## Conclusion

To conclude, apart from the higher salary and working in a city, the most desirable job for the participants in this study would have the Rasmi employment contract, a low workload, adequate workplace facilities, an appropriate work schedule, and 1 to 3 years spent on the assigned job to promote. By sorting the WTP for the attributes of this study, location, work schedule, type of employment contract, workload, expected time spent on the assigned job for promotion to a higher position, and workplace facilities are, respectively, the most important factors that nurses are willing to consider different salaries to change them. These results indicated that nurses are willing to forego net income in exchange for other favorable characteristics of their working environment and conditions. Table 4 shows the amount of monetary value (as the willingness to pay) for each attribute level to indicate how much salary nurses are willing to forego or receive for a certain characteristic of their job. This shows that a variety of actions are accessible to policymakers that can greatly enhance the working conditions for nurses. For example, by creating a universal promotional system for all nurses and setting the baseline to more than 5 years of waiting for promotion, we can give the advantage of promotion in a period of 3 to 5 years (monetary value = 8.35 million IRR) to nurses who work in rural areas. The WTP and the probability of selecting various attributes may help policymakers plan more effectively. For instance, according to the willingness to pay presented in Table 6, recruiting male nurses in rural areas can possibly cost approximately 60 million IRR less than recruiting female nurses. These findings can be applied to the development of employment strategies and policies to attract and retain nurses in a variety of settings (deprived areas, etc.) in Iran.

### Supplementary Information

**Additional file 1.** Questionnaire.

## Data Availability

Contact the corresponding author to obtain the aforementioned data.

## References

[1] Dolea C. Increasing access to health workers in remote and rural areas through improved retention. World Health Organization. 2009. 2–4 p. 23741785

[2] Afzal M, Cometto G, Rosskam E, Sheikh M (2011). Global Health Workforce Alliance: increasing the momentum for health workforce development. Rev Peru Med Exp Salud Publica.

[3] Ross SJ, Polsky D, Sochalski J (2005). Nursing shortages and international nurse migration. Int Nurs Rev.

[4] Mumbauer A, Strauss M, George G, Ngwepe P, Bezuidenhout C, de Vos L (2021). Employment preferences of healthcare workers in South Africa: findings from a discrete choice experiment. PLoS ONE.

[5] Abbaszadeh A, Abdi A (2017). Nursing shortage challenge: a serious threat for the health system: a review study. Community Heal J.

[6] Littlejohn L, Campbell JCM (2012). Nursing shortage: a comparative analysis. Int J Nurs.

[7] Shahraki M (2020). The determinants of nursing workforce demand and predicting the number of the required nurses in the public hospitals of Iran (2018–2025). IRAN J Nurs.

[8] Mehrdad R (2009). Health System in Iran. JMAJ.

[9] Kazemi Karyani A, Karami Matin B, Malekian P, Moradi Rotvandi D, Amini S, Delavari S, Soltani S, Rezaei S (2020). Preferences of medical sciences students for work contracts in deprived areas of Iran: a discrete choice experiment analysis. Risk Manag Healthc Policy.

[10] Rockers PC, Jaskiewicz W, Wurts L, Kruk ME, Mgomella GS, Ntalazi F (2012). Preferences for working in rural clinics among trainee health professionals in Uganda: a discrete choice experiment. BMC Health Serv Res.

[11] Vujicic M, Alfano M, Ryan M, Wesseh CS, Brown-Annan J. Policy options to attract nurses to rural Liberia: Evidence from a discrete choice experiment. Health, Nutrition and Population (HNP) Discussion Paper, World Bank Human Development Network (HNP) Discussion Paper, World Bank Human Development Network. 2010. http://www-wds.worldbank.org/external/default/WDSContentServer/WDSP/IB/2010/11/22/000333037_20101122234724/Rendered/PDF/580200WP01PUBL1ses0to0rural0Liberia.pdf.

[12] Rafiei S, Arab M, Rashidian A, Mahmoudi M, Rahimi-Movaghar V (2015). Policy interventions to improve rural retention among neurosurgeons in Iran: a discrete choice experiment. Iran J Neurol.

[13] Inc. SI. Consumer research. In: JMP® 16 Consumer Research Cary, NC: SAS Institute Inc. 2021. p. 105.

[14] De Bekker-Grob EW, Ryan M, Gerard K (2012). Discrete choice experiments in health economics: a review of the literature. Health Econ.

[15] Huicho L, Miranda JJ, Diez-Canseco F, Lema C, Lescano AG, Lagarde M (2012). Job preferences of nurses and midwives for taking up a rural job in Peru: a discrete choice experiment. PLoS ONE.

[16] Helter TM, Boehler CEH (2016). Developing attributes for discrete choice experiments in health: a systematic literature review and case study of alcohol misuse interventions. J Subst Use.

[17] Civil Service Management Law, approved by the Islamic Council of Iran. Islamic Parliament Research Center. 2006. Available from: https://rc.majlis.ir/en.

[18] Huber J, Zwerina K (1996). The importance of utility balance in efficient choice designs. J Mark Res.

[19] Johnson FR, Yang J-C, Reed SD (2019). The internal validity of discrete choice experiment data: a testing tool for quantitative assessments. Value Health..

[20] Johnson R, Orme B. Getting the most from CBC. Sequim: Sawtooth Software Research Paper Series, Sawtooth Software. 2003 Apr.

[21] Ryan M, Kolstad J, Rockers PDC (2006). How to conduct a discrete choice experiment for health workforce recruitment and retention in remote and rural areas: a user guide with case studies. World Health Org CapacityPlus.

[22] Liu S, Li S, Yang R, Liu T, Chen G (2018). Job preferences for medical students in China: a discrete choice experiment. Medicine (Baltimore).

[23] Park B-H, Ko Y (2016). Hospital preferences of nursing students in Korea: a discrete choice experiment approach. Hum Resour Health.

[24] Araujo E, Maeda A. How to recruit and retain health workers in rural and remote areas in developing countries: a guidance note. World Bank. 2013. 40–43.

