# Supplementary material for: Nurses’ job preferences for working in deprived areas in Tehran: a discrete choice experiment
Source: Hum Resour Health. 2023 Nov 27;21:91. doi: 10.1186/s12960-023-00875-9 (PMC10680310; doi:10.1186/s12960-023-00875-9)
Supplement: Supplementary file 1 — Additional file 1. Questionnaire. [file 12960_2023_875_MOESM1_ESM.docx]

**Part 1: Introduction of the Questionnaire**

The present questionnaire is aimed at conducting a dissertation on the subject of assessing nurses' preferences regarding the supply of nursing labor and the factors affecting it in Tehran. We appreciate you assisting us with the data collection. The questionnaire can be finished in approximately 10 minutes.

There are two sections to this questionnaire. In the first part, socioeconomic status and demographic data are gathered, and in the last part, you will be asked to select which of two employment possibilities you believe is preferable. The information obtained from the questionnaire will be kept confidential at Tehran University of Medical Sciences. This study has ethical approval from the Research Ethics Committee of Tehran University of Medical Sciences, School of Public Health (Ethical code IR.TUMS.SPH.REC.1400.344).

Thank you for your participation.

I agree with the aforementioned terms and conditions.

**Part 2: General questions**

1. How old are you? (in years)

[Numeric input]

2. What is your gender?

Male

Female

3. What is your marital status?

Single

Married

The spouse is deceased /divorced

4. How many children do you have?

No child

One child

Two children

Three children

More than three children

5. Are you the head of the household?

Yes

No

6. How many people are in your household?

One person

Two people

Three people

Four people

Five people

More than five people

7. What is your last degree?

Bachelor's degree

Master's degree

PhD or higher

8. Where do you work?

Public hospital

Private hospital

Health house

Public clinic

Private clinic

9. How many years of work experience do you have? (in years)

[Numeric input]

10. Do you have experience working in deprived areas?

Yes

No [If this was chosen, Question 12 would be skipped.]

12. How many years of experience do you have working in deprived areas?

[Numeric input]

13. What is your current job title? 
 Nurse
 Special department nurse (operating room technician, anesthesia, etc.) 
 Head nurse
 Supervisor and higher

14. What is your type of employment contract?

Rasmi

Gharardadi

Peymani

Tarhi

15. What is the total number of working hours per month?

[Numeric input]

16. How much is your monthly income?

Less than 60 million IRR

Between 60 and 80 million IRR

Between 80 and 100 million IRR

Between 100 and 120 million IRR

Between 120 and 150 million IRR

Between 150 and 200 million IRR

More than 200 million IRR

17. How much are your household's monthly expenses?

Between 30 and 50 million IRR

Between 50 and 70 million IRR

Between 70 and 100 million IRR

Between 100 and 150 million IRR

Between 150 and 200 million IRR

More than 200 million IRR

18. If the conditions are available, do you want to emigrate from the country?

Yes

No

19. Subjective social status 
Imagine a ladder that has 10 steps, and the steps represent different classes of society. At the highest level, which is the 10th level, there are people with the most wealth, the highest education, and the best jobs, and at the lowest level, which is the 1st level, there are people with the least wealth, low education, and low-respecting jobs.

Where is your place on this hypothetical ladder from one to ten?

[Numeric input]

**Part 3: Introduction of the choice experiment**

In this section, you can see two job options. The two options are compared in terms of income, workload, contract status, type of workplace, etc. in a table.
 Please choose the option that you think is better. 
It should be noted that there are no right or wrong answers, and only the individual's preference is measured. The table below is displayed as a guide and benchmark for better decision-making.

| Workload | Low (in the special, 2 patients per nurse, and in the normal department, 5 to 7 patients per nurse).  Moderate (in the special department, 3 patients per nurse, and in the normal department, 8 to 9 patients per nurse).  Heavy (in the special department, 4 patients per nurse, and in the normal department, 10 patients or more per nurse). |
| --- | --- |
| Workplace facilities | Inadequate (No free transportation, no free meals on shift, break room without amenities).  Adequate (Free transportation is available, Free meals on shift, break room with standard amenities like microwave and refrigerator). |
| Work schedule | Appropriate (regular shifts, and the full cooperation of the supervisor in monthly planning according to individual conditions).  Inappropriate (Irregular shifts in the month, the presence of night shifts, working on holidays, and the lack of cooperation of the supervisor in the monthly planning according to the individual). |

**Part 4: Choice experiment**

In this part, the participant must select one preferable profile among those available (options).

**Survey 1 (block 1)**

Scenario 1

| Attribute | Profile 1 | Profile 2 |
| --- | --- | --- |
| Monthly salary | 150 million IRR | 100 million IRR |
| Location | Rural (Deprived Area) | City |
| Type of employment contract | Rasmi | Peymani |
| Workload | Moderate | Heavy |
| Type of facility | Hospital | Health House |
| Workplace facilities | Adequate | Inadequate |
| Work schedule | Inappropriate | Appropriate |
| Expected time spent on the assigned job for promotion to a higher position | 1 to 3 years | 3 to 5 years |

Scenario 2

| Attribute | Profile 1 | Profile 2 |
| --- | --- | --- |
| Monthly salary | 200 million IRR | 150 million IRR |
| Location | City | City |
| Type of employment contract | Rasmi | Peymani |
| Workload | Moderate | Heavy |
| Type of facility | Health House | Clinic |
| Workplace facilities | Inadequate | Adequate |
| Work schedule | Appropriate | Inappropriate |
| Expected time spent on the assigned job for promotion to a higher position | 3 to 5 years | 1 to 3 years |

Scenario 3

| Attribute | Profile 1 | Profile 2 |
| --- | --- | --- |
| Monthly salary | 150 million IRR | 100 million IRR |
| Location | Rural (Deprived Area) | Rural (Deprived Area) |
| Type of employment contract | Peymani | Gharardadi |
| Workload | Heavy | Moderate |
| Type of facility | Health House | Hospital |
| Workplace facilities | Adequate | Inadequate |
| Work schedule | Inappropriate | Appropriate |
| Expected time spent on the assigned job for promotion to a higher position | More than 5 years | 3 to 5 years |

Scenario 4

| Attribute | Profile 1 | Profile 2 |
| --- | --- | --- |
| Monthly salary | 200 million IRR | 100 million IRR |
| Location | Rural (Deprived Area) | Rural (Deprived Area) |
| Type of employment contract | Rasmi | Peymani |
| Workload | Heavy | Low |
| Type of facility | Clinic | Hospital |
| Workplace facilities | Inadequate | Adequate |
| Work schedule | Inappropriate | Appropriate |
| Expected time spent on the assigned job for promotion to a higher position | 3 to 5 years | 1 to 3 years |

Scenario 5

| Attribute | Profile 1 | Profile 2 |
| --- | --- | --- |
| Monthly salary | 150 million IRR | 200 million IRR |
| Location | Rural (Deprived Area) | City |
| Type of employment contract | Rasmi | Gharardadi |
| Workload | Low | Moderate |
| Type of facility | Health House | Clinic |
| Workplace facilities | Adequate | Inadequate |
| Work schedule | Appropriate | Inappropriate |
| Expected time spent on the assigned job for promotion to a higher position | 3 to 5 years | More than 5 years |

Scenario 6

| Attribute | Profile 1 | Profile 2 |
| --- | --- | --- |
| Monthly salary | 150 million IRR | 150 million IRR |
| Location | City | City |
| Type of employment contract | Peymani | Gharardadi |
| Workload | Moderate | Low |
| Type of facility | Clinic | Hospital |
| Workplace facilities | Inadequate | Adequate |
| Work schedule | Appropriate | Inappropriate |
| Expected time spent on the assigned job for promotion to a higher position | 1 to 3 years | 3 to 5 years |

**Survey 2 (block 2)**

Scenario 7

| Attribute | Profile 1 | Profile 2 |
| --- | --- | --- |
| Monthly salary | 200 million IRR | 150 million IRR |
| Location | City | City |
| Type of employment contract | Peymani | Rasmi |
| Workload | Moderate | Low |
| Type of facility | Hospital | Clinic |
| Workplace facilities | Inadequate | Inadequate |
| Work schedule | Appropriate | Inappropriate |
| Expected time spent on the assigned job for promotion to a higher position | 1 to 3 years | 3 to 5 years |

Scenario 8

| Attribute | Profile 1 | Profile 2 |
| --- | --- | --- |
| Monthly salary | 200 million IRR | 100 million IRR |
| Location | Rural (Deprived Area) | City |
| Type of employment contract | Peymani | Rasmi |
| Workload | Heavy | Low |
| Type of facility | Clinic | Health House |
| Workplace facilities | Adequate | Inadequate |
| Work schedule | Appropriate | Inappropriate |
| Expected time spent on the assigned job for promotion to a higher position | 3 to 5 years | 1 to 3 years |

Scenario 9

| Attribute | Profile 1 | Profile 2 |
| --- | --- | --- |
| Monthly salary | 100 million IRR | 150 million IRR |
| Location | Rural (Deprived Area) | Rural (Deprived Area) |
| Type of employment contract | Peymani | Gharardadi |
| Workload | Low | Moderate |
| Type of facility | Clinic | Hospital |
| Workplace facilities | Inadequate | Adequate |
| Work schedule | Inappropriate | Appropriate |
| Expected time spent on the assigned job for promotion to a higher position | 1 to 3 years | More than 5 years |

Scenario 10

| Attribute | Profile 1 | Profile 2 |
| --- | --- | --- |
| Monthly salary | 200 million IRR | 150 million IRR |
| Location | City | Rural (Deprived Area) |
| Type of employment contract | Peymani | Gharardadi |
| Workload | Moderate | Low |
| Type of facility | Health House | Clinic |
| Workplace facilities | Adequate | Inadequate |
| Work schedule | Inappropriate | Appropriate |
| Expected time spent on the assigned job for promotion to a higher position | More than 5 years | 1 to 3 years |

Scenario 11

| Attribute | Profile 1 | Profile 2 |
| --- | --- | --- |
| Monthly salary | 200 million IRR | 150 million IRR |
| Location | City | City |
| Type of employment contract | Gharardadi | Peymani |
| Workload | Low | Moderate |
| Type of facility | Health House | Hospital |
| Workplace facilities | Adequate | Inadequate |
| Work schedule | Appropriate | Inappropriate |
| Expected time spent on the assigned job for promotion to a higher position | 1 to 3 years | 3 to 5 years |

Scenario 12

| Attribute | Profile 1 | Profile 2 |
| --- | --- | --- |
| Monthly salary | 200 million IRR | 150 million IRR |
| Location | City | City |
| Type of employment contract | Rasmi | Peymani |
| Workload | Heavy | Moderate |
| Type of facility | Hospital | Clinic |
| Workplace facilities | Inadequate | Adequate |
| Work schedule | Inappropriate | Appropriate |
| Expected time spent on the assigned job for promotion to a higher position | 1 to 3 years | 3 to 5 years |

**Survey 3 (block 3)**

Scenario 13

| Attribute | Profile 1 | Profile 2 |
| --- | --- | --- |
| Monthly salary | 150 million IRR | 100 million IRR |
| Location | Rural (Deprived Area) | City |
| Type of employment contract | Gharadadi | Peymani |
| Workload | Moderate | Heavy |
| Type of facility | Health House | Hospital |
| Workplace facilities | Adequate | Inadequate |
| Work schedule | Inappropriate | Appropriate |
| Expected time spent on the assigned job for promotion to a higher position | 1 to 3 years | More than 5 years |

Scenario 14

| Attribute | Profile 1 | Profile 2 |
| --- | --- | --- |
| Monthly salary | 110 million IRR | 100 million IRR |
| Location | City | Rural (Deprived Area) |
| Type of employment contract | Gharardadi | Rasmi |
| Workload | Heavy | Low |
| Type of facility | Health House | Clinic |
| Workplace facilities | Inadequate | Adequate |
| Work schedule | Appropriate | Inappropriate |
| Expected time spent on the assigned job for promotion to a higher position | 1 to 3 years | 3 to 5 years |

Scenario 15

| Attribute | Profile 1 | Profile 2 |
| --- | --- | --- |
| Monthly salary | 100 million IRR | 200 million IRR |
| Location | City | Rural (Deprived Area) |
| Type of employment contract | Rasmi | Peymani |
| Workload | Moderate | Low |
| Type of facility | Clinic | Health House |
| Workplace facilities | Adequate | Inadequate |
| Work schedule | Inappropriate | Inappropriate |
| Expected time spent on the assigned job for promotion to a higher position | More than 5 years | 3 to 5 years |

Scenario 16

| Attribute | Profile 1 | Profile 2 |
| --- | --- | --- |
| Monthly salary | 100 million IRR | 150 million IRR |
| Location | City | Rural (Deprived Area) |
| Type of employment contract | Gharardadi | Peymani |
| Workload | Moderate | Low |
| Type of facility | Clinic | Hospital |
| Workplace facilities | Adequate | Inadequate |
| Work schedule | Inappropriate | Appropriate |
| Expected time spent on the assigned job for promotion to a higher position | 3 to 5 years | More than 5 years |

Scenario 17

| Attribute | Profile 1 | Profile 2 |
| --- | --- | --- |
| Monthly salary | 150 million IRR | 200 million IRR |
| Location | City | Rural (Deprived Area) |
| Type of employment contract | Gharardadi | Gharardadi |
| Workload | Heavy | Moderate |
| Type of facility | Hospital | Clinic |
| Workplace facilities | Inadequate | Adequate |
| Work schedule | Inappropriate | Appropriate |
| Expected time spent on the assigned job for promotion to a higher position | 3 to 5 years | More than 5 years |

Scenario 18

| Attribute | Profile 1 | Profile 2 |
| --- | --- | --- |
| Monthly salary | 100 million IRR | 100 million IRR |
| Location | Rural (Deprived Area) | Rural (Deprived Area) |
| Type of employment contract | Rasmi | Gharardadi |
| Workload | Heavy | Low |
| Type of facility | Hospital | Clinic |
| Workplace facilities | Adequate | Inadequate |
| Work schedule | Appropriate | Inappropriate |
| Expected time spent on the assigned job for promotion to a higher position | 1 to 3 years | More than 5 years |

Internal validity testing scenario (This is the first scenario of all surveys)

| Attribute | Profile 1 | Profile 2 |
| --- | --- | --- |
| Monthly salary | 10 million IRR | 20 million IRR |
| Location | Rural (Deprived Area) | City |
| Type of employment contract | Gharadadi | Rasmi |
| Workload | Heavy | Low |
| Type of facility | Health House | Hospital |
| Workplace facilities | Inadequate | Adequate |
| Work schedule | Inappropriate | Appropriate |
| Expected time spent on the assigned job for promotion to a higher position | More than 5 years | 1 to 3 years |
